# Increased EMRSA-15 health-care worker colonization demonstrated in retrospective review of EMRSA hospital outbreaks

**DOI:** 10.1186/2047-2994-3-7

**Published:** 2014-03-03

**Authors:** Julie Hart, Keryn J Christiansen, Rosie Lee, Christopher H Heath, Geoffrey W Coombs, J Owen Robinson

**Affiliations:** 1 Department of Microbiology and Infectious Diseases, PathWest Laboratory Medicine, Royal Perth Hospital, Perth, Western Australia, Australia; 2 Australian Collaborating Centre for Enterococcus and Staphylococcus Species Typing and Research, Curtin University, Perth, Western Australia, Australia; 3 School of Medicine and Pharmacology, University of Western Australia, Royal Perth Hospital, Perth, WA, Australia

**Keywords:** Methicillin resistant *Staphylococcus aureus*, Outbreak, Health care workers, EMRSA-15, Aus2/3-EMRSA

## Abstract

**Background:**

Health care worker (HCW) colonization with methicillin resistant *Staphylococcus aureus* (MRSA) is a documented cause of hospital outbreaks and contributes to ongoing transmission. At Royal Perth Hospital (RPH) it had been anecdotally noted that the increasing prevalence of EMRSA-15 appeared to be associated with increased HCW colonization compared with Aus2/3-EMRSA. Hence we compared HCW colonization rates during outbreaks of EMRSA-15 and Aus2/3-EMRSA at a single institution.

**Methods:**

We performed a retrospective review of EMRSA-15 and Aus2/3-EMRSA outbreaks from 2000–2009 at RPH, a quaternary hospital in Western Australia. Outbreak files were reviewed and relevant data extracted.

**Results:**

Ten EMRSA-15 outbreaks were compared with seven Aus2/3 outbreaks. The number of patients colonized was similar between EMRSA-15 and Aus2/3-EMRSA outbreaks (median 7 [range 3–20] and 11 [5–26], respectively; *P* = 0.07) but the number of HCWs colonized was significantly higher in EMRSA-15 outbreaks compared to Aus2/3-EMRSA outbreaks (median 4 [range 0–15] and 2 [1-3], respectively; *P* = 0.013). The percentage of HCWs colonized was also higher in EMRSA-15 outbreaks versus Aus2/3-EMRSA outbreaks (median 3.4% [range 0–5.5%] and 0.81% [0.56–2.2%], respectively; *P* = 0.013).

**Conclusions:**

This study demonstrates a higher level of HCW colonization during EMRSA-15 outbreaks compared with Aus2/3-EMRSA outbreaks. This finding suggests that MRSA vary in their ability to colonize HCWs and contribute to outbreaks. MRSA type should be determined during outbreaks and future research should investigate the mechanisms by which EMRSA-15 contributes to increased HCW colonization.

## Background

Methicillin resistant *Staphylococcus aureus* (MRSA) is an important cause of hospital-acquired infection and has a high mortality rate. At Royal Perth Hospital (RPH) a 10-year retrospective study demonstrated a 30-day mortality rate of 27.5% for hospital associated MRSA bacteraemia [1], similar to an Australasian study, in which 30-day mortality was 30% [2].

Healthcare-associated MRSA (HA-MRSA) can cause hospital outbreaks and rapidly replace other *S. aureus* strains [3]. The most prevalent HA-MRSA clones in Australia are ST22-MRSA-IV (also known as epidemic MRSA (EMRSA)-15), and ST239-MRSA-III (Aus2/3-EMRSA or EMRSA-1), which accounted for 45.9% and 49.3% of all HA-MRSA isolated in the 2011 Australian Group on Antimicrobial Resistance national antimicrobial surveillance on hospital-onset *S. aureus* infections [4].

Healthcare worker (HCW) involvement in MRSA transmission has been summarised in a review of 127 outbreak studies [5]. In this review, 4.6% of 33,318 HCWs were MRSA colonized, with a higher prevalence in the Australian/New Zealand studies (9.7%). A point prevalence study performed at RPH showed a prevalence of MRSA colonisation of only 3.4% among 1,542 HCWs screened, and the prevalence was higher in HCWs working on high risk wards (defined as isolation wards where known MRSA colonised patients were admitted and geriatric wards) – 10.7% versus 2.5% on low risk (all other) wards (*P* < 0.01) [6]. MRSA transmission from HCWs to patients has been well documented. In a 10-year retrospective Dutch study, 13/17 MRSA outbreaks involved HCWs, with at least four of the outbreaks implicating HCWs as the index case [7]. A review showed that 63/68 (93%) studies that undertook patient and HCW MRSA genotyping demonstrated isolates were likely clonally related [5].

In Western Australia (WA) a “search and destroy” MRSA management policy has successfully controlled nosocomial spread of HA-MRSA [8]. HCW MRSA colonization contributes to the cause and maintenance of hospital MRSA outbreaks, hence better understanding of the factors that contribute to HCW MRSA colonization will lead to improved control of MRSA outbreaks. At RPH it had been noted anecdotally that the increasing prevalence of EMRSA-15 appeared to be associated with increased HCW colonization compared to that seen with other MRSA strains. We therefore performed a retrospective study of EMRSA-15 and Aus2/3-EMRSA outbreaks from 2000–2009 to determine the colonisation rate in HCWs according to the MRSA strain.

## Methods

### Setting

A retrospective review was performed on MRSA outbreaks recorded by the infection control team at RPH from 1st January 2000 to 31st December 2009. RPH is a quaternary 724-bed hospital, with approximately 7000 staff members. EMRSA are not endemic at RPH. As a retrospective non-interventional study Ethics Committee review was waived.

### Population screened

WA MRSA management policy mandates screening on admission to hospital of long-term care facility residents and patients who have been hospitalized or employed in a hospital outside WA, in the preceding year. Screening is also performed at RPH on patients admitted for elective cardiothoracic or orthopaedic surgery or to intensive care or bone marrow transplant units. Additionally, patients epidemiologically linked to single strain outbreaks (defined under “Outbreak Definition”) are also screened. HCWs who have been hospitalised or employed in a hospital outside of WA in the preceding 12 months also have mandatory screening. HCWs working on wards with single strain outbreaks are also screened (detailed under “Outbreak Definition”). The policy also includes MRSA decolonization, which has been shown to assist in preventing and controlling nosocomial transmission of MRSA [5].

### Microbiology methods

During the study period patient screening included swabbing anterior nares and any broken skin. Prior to 2003, throat and perineum swabs were also collected. Screening of HCWs included swabbing anterior nares, throat and all broken skin areas. From 2008 onwards the nose and throat specimens were tested using the BD GeneOhm™ MRSA Assay (instead of culture only which was performed prior to 2008) and positives were confirmed by culture. Throughout the study period culture only was performed on swabs collected from other sites; briefly, swabs were inoculated onto MRSA selective solid media and into MRSA enrichment broth which was subcultured after 20 hours incubation. Antimicrobial susceptibility testing was performed by either disc diffusion on Mueller-Hinton agar (CLSI criteria), the Vitek2 ® AST-P579 card (bioMérieux) or E-test ® (bioMérieux). HA-EMRSA strains were characterised by the Australian Collaborating Centre for *Enterococcus* and S*taphylococcus* Species (*ACCESS*) Typing and Research as previously described [1].

### Outbreak definition

An outbreak was defined when three or more patients with the same MRSA strain were temporally linked to the same ward. From 1993 to 2004, the isolation of MRSA from a patient prompted all room contacts to be screened. If a second patient was identified screening was extended to all patients on the ward. If a third patient was identified with the same MRSA strain, a second ward screen was performed, including all HCWs (medical, nursing, allied health and patient care assistants [PCAs]) associated with the ward. From 2004 onwards HCW screening was performed using the same protocol if the outbreak was EMRSA-15. For non-EMRSA-15 investigations, HCW screening was only performed if more than three patients were identified with the same strain. For the whole study period, follow-up screening ceased after two negative ward screens, usually performed three to four weeks apart. Commencement and resolution of an outbreak were defined as the date when the first and last MRSA colonized patient or HCW were identified.

### Outbreak control

Patients were isolated in single rooms with contact precautions. Decolonization occurred if patients did not have any wounds or invasive devices and involved nasal mupirocin 2%, three times per day, for 10 days), whole-body antisepsis (3% hexachlorophene emulsion once daily, for 10 days and 20% cetrimide shampoo three times per week). Decolonization of HCWs was the same and HCWs on decolonization treatment were able to return to work immediately. Decolonization was commenced without repeating the initial screening swabs, hence transient or persistent colonization could not be distinguished. Systemic antibiotics were prescribed if patients or HCWs had MRSA throat carriage or remained persistently colonized post decolonization treatment. During an outbreak two-step cleaning and disinfection (phenolic 1:64) of the environment was employed.

### Measures and statistical analysis

For each EMRSA-15 or Aus2/3-EMRSA outbreak the following information was collected: patient demographic data, outbreak location and duration and the number of patients and HCWs screened. Statistical analysis was performed using IBM SPSS version 19. Means were compared using the Student’s *t*-test or the Wilcoxon non-parametric test, where appropriate. Percentages were compared with the Pearson’s χ^2^ or Fischer’s exact test. *P* values of < 0.05 were considered significant.

## Results

**Figure 1:**
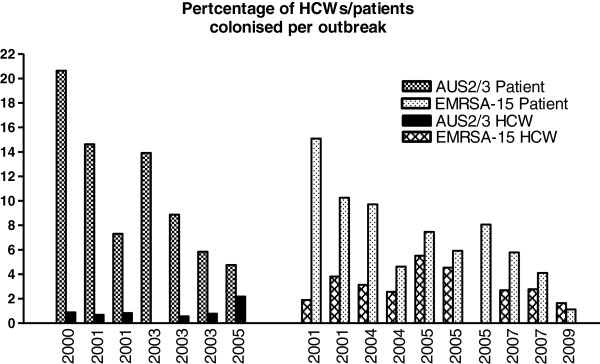
Percentage patients and HCWs colonized by outbreak strain and year (multiple outbreaks occurred in some years).

**Figure 2:**
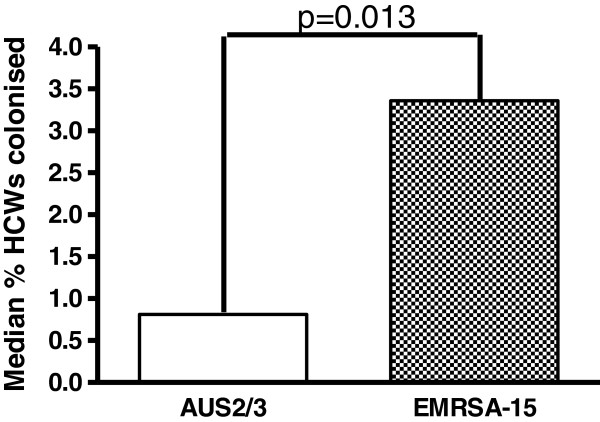
Comparison of median patient and HCW colonization rates for all EMRSA-15 and Aus2/3-EMRSA outbreaks.

**Figure 3:**
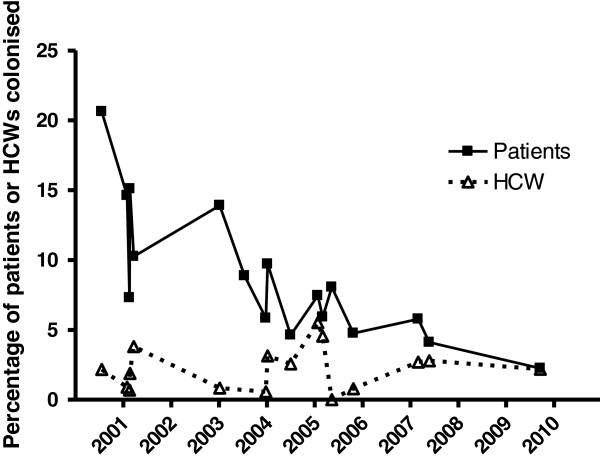
Proportion of patients and HCWs colonized during outbreaks of EMRSA-15 and Aus2/3-EMRSA 2000–2009.

We detected ten EMRSA-15 and seven Aus2/3-EMRSA outbreaks and median outbreak durations were comparable (50 and 52 days, respectively). The number of patients and HCWs screened was similar between EMRSA-15 and Aus2/3-EMRSA outbreaks (Table 1). The number and percentage of patients colonized with EMRSA-15 and Aus2/3-EMRSA were equivalent (Figure 1, Table 1). Both the number and percentage of HCWs colonized with EMRSA-15 were significantly higher compared to HCWs colonized with Aus2/3-EMRSA; median 4 [range 0–15] and 2 [1-3]; *P* = 0.013 (Table 1) and median 3.4% (range 0–5.5%) and 0.81% (0.56–2.2%); *P* = 0.013 (Table 1, Figures 2 and 3). In one Aus2/3-EMRSA outbreak the number of HCWs screened was not recorded. The percentage of HCWs who could not be contacted or who declined screening was similar during both EMRSA-15 and Aus2/3-EMRSA outbreaks (19% and 13%). Over the study period the proportion of colonized patients per outbreak declined, but the proportion of HCWs colonized remained stable (Figure 3). Nursing staff comprised the majority of colonized HCWs (40 of 64 [63%] whose role was known), followed by PCAs (17 of 64 [27%]). No colonized agency nursing staff were detected, but they were screened much less frequently than permanent nursing staff (160 of 312 [51%] agency staff were not screened compared with 37 of 304 [12%] permanent nursing staff).

**Table 1:** Comparison of EMRSA-15 and Aus2/3-EMRSA outbreaks

	**EMRSA-15**	**Aus2/3**	***P*** **value**
Number of outbreaks	10	7	NA
Median duration of outbreaks (range)	50 days (22–128)	52 days (20–392)	0.69
Median number of patients screened (range)	106 (39–338)	124 (103–137)	0.59
Median number of HCWs screened (range)	125 (72–330)	159 (120–288)	0.30
Median number of patients colonized (range)	7 (3–20)	11 (5–26)	0.07
Median percent of patients colonized (range)	6.69 (1.12-15.09)	8.87 (4.76-20.63)	0.27
Median number of HCWs colonized (range)	4 (0–15)	2 (1–3)	0.013
Median percent of HCW colonized (range)	3.36 (0–5.52)	0.81 (0.56-2.17)	0.013

## Discussion

To our knowledge this is the first study to show a significantly higher prevalence of HCW colonization during EMRSA-15 outbreaks compared to Aus2/3-EMRSA outbreaks. Few prior studies provide details of the EMRSA strain type and a comparison of the prevalence of both patient and HCW colonization. Our study suggests MRSA strains vary in their ability to colonize HCWs and contribute to outbreaks. EMRSA-15 has become the predominant HA-MRSA in Australia and many other countries [9], so our results have implications for daily practice. We therefore believe MRSA typing should be determined during outbreaks to influence decisions on HCW screening and decolonisation and should be reported in infection control publications, to better determine the influence of MRSA type on outbreak characteristics and control.

A German study comparing MRSA carriage between staff and residents at a nursing home found more carriage of a PVL-positive strain (7.6% of 197 residents and 5.8% of 104 staff) compared with a multidrug resistant MRSA strain (3.0% of 197 residents and 1% of 104 staff) [10]. Although this study was not specifically designed to compare patient and HCW MRSA carriage rates based on ST type, it also suggests that HCW carriage rates vary between EMRSA ST types. We could not find any other studies specifically designed to address the question of variable HCW MRSA carriage rates by sequence type.

The prevalence of HCW colonization (3.4% EMRSA-15 and 0.81% Aus2/3-EMRSA) in our study was lower than other published Australia and New Zealand studies [5]. This is probably attributable to our longstanding state-wide, “search and destroy” MRSA management policy that includes screening of high risk patients, aggressive outbreak management policies and strict isolation and contact precautions for patients with epidemic HA-MRSA colonization. The decrease in proportion of patients colonized over the study period suggests measures introduced to control MRSA transmission have been successful at RPH. However the proportion of HCWs colonized remained stable over the same period, suggesting control measures other than those designed to limit transmission via HCWs are responsible for the reduced proportion of colonized patients. This implies hand hygiene and methods to prevent transmission to HCWs should remain an ongoing focus of outbreak management. At RPH hand hygiene compliance rates were poor when a formal hand hygiene auditing program was first introduced in February 2008 – 40% across all HCW groups (personal communication, Rosie Lee).

The majority of colonized HCWs in this study were nursing staff, followed by PCAs. These two groups should remain a focus for education in outbreak management plans. A MRSA HCW prevalence study performed at RPH also showed highest levels of colonization in HCWs most in contact with patients – 6.8% of patient care assistants and 5.2% of nursing staff, compared with 0.7% of doctors [5]. No agency nursing staff in our study were colonized despite representing a large proportion of nursing staff, possibly as a result of the low numbers who were available or agreed to be screened (results not presented). This finding may have introduced bias and suggests that strategies need to be developed to enable a larger proportion of agency staff to be screened to determine if they are an unidentified reservoir of MRSA transmission, particularly as they more commonly work at multiple hospitals and residential care facilities. All HCWs from outside of WA undergo pre-employment MRSA screening and decolonization, so any increase in HCWs transferring from EMRSA-15 endemic hospitals is unlikely to explain the increased HCW EMRSA-15 colonization rates.

Potential limitations of this study are the retrospective design and the small numbers of outbreaks and low colonization rates, but despite this our findings were statistically significant. Microbiology methods changed over the study period, which may have increased MRSA detection rates in the latter years of the study when EMRSA-15 predominated. Aus2/3-EMRSA has been noted anecdotally at RPH to preferentially colonize the perineum. The perineum was not screened in HCWs, so if this association is true then Aus2/3-EMRSA colonisation of HCWs may have been underestimated. A significant number of HCWs were not contactable or declined MRSA screening during each outbreak, which may have introduced bias, although the percentage of HCWs not screened was similar during EMRSA-15 and Aus2/3-EMRSA outbreaks. *S. aureus* carriage can be transient and HCWs in our institution are given decolonization therapy prior to any repeat screening, so this could have created biases within our dataset. Transient MRSA colonization rates in HCWs (32%) are known from a prior prevalence study at RPH [6]. Changes to outbreak investigation policy in 2004 meant non-EMRSA-15 outbreaks were investigated only when a fourth patient was found colonized which may have caused a delay in HCW screening and less instigation of HCW screening during Aus2/3-EMRSA outbreaks. However, only one Aus2/3-EMRSA outbreak involved HCW screening after this guideline change, consequently this is unlikely to have affected the results. Increased awareness of MRSA amongst HCWs and improved infection control team EMRSA outbreak management over the decade should have reduced HCW colonization in the later stages of the study when EMRSA-15 became the predominant outbreak strain, nonetheless our HCW colonization rates remained higher during EMRSA-15 outbreaks.

The reasons for increased EMRSA-15 colonization of HCWs are largely unknown. Biologically plausible explanations include that EMRSA-15 carries fewer resistant traits, a smaller SCC*mec* element and “core” genome, together with the acquisition of additional accessory genomic elements may have conferred to this particular clone higher epidemicity and growth rate, greater biofilm formation, enhanced capacity for dissemination and invasion and ability to persist that might explain its greater success and fitness [11]. Therefore, potential factors promoting colonization of HCWs by EMRSA-15 includes its preferential colonization of sites on patients likely to contaminate HCWs (e.g. skin or wounds, in preference to perineum), increased transmission into the environment, ability to adhere to skin and environmental surfaces and replication ability. The mechanisms by which EMRSA-15 leads to higher HCW colonization rates should be of foremost importance in future research studies.

## Conclusions

This is the first study to demonstrate two different HA-MRSA strains (EMRSA-15 and Aus2/3-EMRSA) have differential HCW colonization rates during outbreaks within the same institution. This suggests EMRSA strains vary in their ability to colonize HCWs. This study highlights the importance of characterizing EMRSA strains to determine the likely source of the outbreak and to assist with EMRSA outbreak control. EMRSA type should be determined during outbreaks and future research should investigate the mechanisms by which EMRSA-15 is associated with increased HCW colonization.

## Competing interest

All authors report no conflicts of interest relevant to this article.

## Authors’ contributions

JH collected, analysed and interpreted the data and wrote the manuscript. KC conceived of and participated in the design of the study, RL participated in the design and data collection, CH participated in substantial critical intellectual revision of the manuscript, GC participated in the design and critical intellectual revision of the manuscript and OR helped conceive, design, analyse, interpret and critically revise the manuscript. All authors read, reviewed and provided feedback on the final manuscript.

## References

[B1] Robinson JO Pearson JC Christiansen KJ Coombs GW Murray RJ Community-associated versus healthcare-associated methicillin-resistant *Staphylococcus aureus* bacteraemia: a 10-year retrospective review Eur J Clin Microbiol Infect Dis 2009 3 353 361 10.1007/s10096-008-0632-1 18850122

[B2] Turnidge JD Kotsanas D Munckhof W Roberts S Bennett CM Nimmo GR Coombs GW Murray RJ Howden B Johnson PD Dowling K Australia New Zealand Cooperative on Outcomes in Staphylococcal Sepsis *Staphylococcus aureus* bacteraemia: a major cause of mortality in Australia and New Zealand Med J Aust 2009 3 368 373 19807625 10.5694/j.1326-5377.2009.tb02841.x

[B3] Albrecht N Jatzwauk L Slickers P Ehricht R Monecke S Clonal replacement of methicillin-resistant *Staphylococcus aureus* strains in a German university hospital PLoS One 2011 3 11 28189 10.1371/journal.pone.0028189 PMC3227659 22140542

[B4] Coombs GW Pearson JC Nimmo GR Collignon PJ Bell JM McLaws ML Christiansen KJ Turnidge JD Antimicrobial susceptibility of *Staphylococcus aureus* and molecular epidemiology of meticillin resistant *S. aureus* isolated from Australian hospital inpatients: report from the Australian group on antimicrobial resistance 2011 *Staphylococcus aureus* surveillance programme J Global Antimicrob Resin press 10.1016/j.jgar.2013.04.005 27873625

[B5] Albrich WC Harbarth S Health-care workers: source, vector, or victim of MRSA? Lancet Infect Dis 2008 3 289 301 10.1016/S1473-3099(08)70097-5 18471774

[B6] Verwer PE Robinson JO Coombs GW Wijesuriya T Murray RJ Verbrugh HA Riley T Nouwen JL Christiansen KJ Prevalence of nasal methicillin-resistant *Staphylococcus aureus* colonization in healthcare workers in a Western Australian acute care hospital Eur J Clin Microbiol Infect Dis 2012 3 1067 1072 10.1007/s10096-011-1408-6 21909648

[B7] Blok HE Troelstra A Kamp-Hopmans TE Gigengack-Baars AC Vandenbroucke-Grauls CM Weersink AJ Verhoef J Mascini EM Role of healthcare workers in outbreaks of methicillin-resistant *Staphylococcus aureus*: a 10-year evaluation from a Dutch university hospital Infect Control Hosp Epidemiol 2003 3 679 685 10.1086/502275 14510251

[B8] Coombs GW Van Gessel H Pearson JC Godsell MR O'Brien FG Christiansen KJ Controlling a multicenter outbreak involving the New York/Japan methicillin-resistant *Staphylococcus aureus* clone Infect Control Hosp Epidemiol 2007 3 845 852 10.1086/518726 17564988

[B9] Monecke S Coombs G Shore AC Coleman DC Akpaka P Borg M Chow H Ip M Jatzwauk L Jonas D Kadlec K Kearns A Laurent F O'Brien FG Pearson J Ruppelt A Schwarz S Scicluna E Slickers P Tan HL Weber S Ehricht R A field guide to pandemic, epidemic and sporadic clones of methicillin-resistant Staphylococcus aureus PLoS One 2011 3 4 e17936 10.1371/journal.pone.0017936 21494333 PMC3071808

[B10] Raab U Kahlau D Wagenlehner F Reischl U Ehrenstein V Lehn N Holler C Linde HJ Prevalence of and risk factors for carriage of Panton-Valentine leukocidin-positive methicillin-resistant *Staphylococcus aureus* among residents and staff of a German nursing home Infect Control Hosp Epidemiol 2006 3 208 211 10.1086/500629 16465643

[B11] Holden M Hsu LY Kurt K Weinert LA Mather AE Harris SR Strommenger B Layer F Witte W de Lencastre H Skov R Westh H Zemlicková H Coombs G Kearns AM Hill RL Edgeworth J Gould I Gant V Cooke J Edwards GF McAdam PR Templeton KE McCann A Zhou Z Castillo-Ramírez S Feil EJ Hudson LO Enright MC Balloux F A genomic portrait of the emergence, evolution, and global spread of a methicillin-resistant *Staphylococcus aureus* pandemic Genome Res 2013 3 653 664 10.1101/gr.147710.112 23299977 PMC3613582

